# Progress Toward the Elimination of Vertical HIV Transmission in Nepal: A Retrospective Cohort Study

**DOI:** 10.1007/s10900-025-01474-6

**Published:** 2025-07-01

**Authors:** Upendra Shrestha, Lok Raj Pandey, Man Bahadur KC, Ali Mirzazadeh, Keshab Deuba

**Affiliations:** 1 Public Health Professional, Kathmandu, Nepal; 2 National Center for AIDS and STD Control, Kathmandu, Nepal; 3 Department of Epidemiology and Biostatistics, University of California , San Francisco, CA USA; 4 Public Health and Environment Research Centre (PERC), Lalitpur, Nepal; 5 Department of Global Public Health, Karolinska Institute, Stockholm, Sweden; 6 Centre for International Health (CIH), Department of Global Public Health and Primary Care, University of Bergen, Bergen, Norway

**Keywords:** Vertical HIV Transmission, Antiretroviral Therapy, Pregnant Women Living with HIV, Infants Living with HIV, Nepal

## Abstract

Despite global advancements, pregnant women living with HIV in Nepal remain at risk for vertical transmission. This study examined demographic and clinical characteristics, antiretroviral therapy (ART) retention, and transmission outcomes among this population. A retrospective cohort analysis was conducted using data from Nepal’s national electronic HIV register, including 322 women who became pregnant between 2020 and 2023. We analyzed sociodemographic profiles, clinical status at diagnosis, ART initiation timing, retention rates at 6, 12, and 24 months, and infant HIV status. The mean age was 26.9 years; over half (56.2%) were aged 25–39 years. Nearly 40% were illiterate, 87.6% unemployed, and 66.8% reported unsafe sexual behavior as the mode of HIV transmission. Over half (58.1%) were diagnosed prior to pregnancy, and 75.5% were in WHO Stage 1. ART began on the same day in 34.8% and within a week in 40.1%, with 56.8% already on ART during pregnancy. Retention was high: 96.9% at 6 months, 94.8% at 12 months, and 96.0% at 24 months. Vertical transmission occurred in 4.3% of pregnancies. Higher transmission rates were observed among younger mothers (6.9%), Dalit women (11.5%), those in advanced HIV stages (11.1%), with delayed ART initiation (8.6%), high viral loads (13.3%), and home deliveries (17.6%). In Nepal, approximately 1 in 23 infants born to women living with HIV still acquire the infection. Strengthening early diagnosis, improving ART uptake, and addressing disparities in care especially among high-risk groups are essential to eliminating vertical transmission and improving maternal and child health outcomes.

## Introduction

The World Health Organization (WHO) reported that in 2023, approximately 1.2 million women and girls living with HIV became pregnant globally [1]. Without treatment interventions, the rate of HIV transmission from a mother living with HIV to her child ranges from 15 to 30% during pregnancy and delivery, increasing by an additional 5–15% through breastfeeding [2]. The women of reproductive age accounted for 44% of all new HIV infections. An estimated 120,000 children (range: 83,000–170,000) acquired new HIV infections globally, while the antiretroviral therapy (ART) coverage among pregnant women living with HIV (PWHIV) was 84% (range: 72–98%) [1–3]. In the Asia-Pacific region, an estimated 6.7 million (range: 6.1 million–7.5 million) people were living with HIV (PLHIV) in 2023, with 120,000 (range: 100,000–140,000) new infections occurring annually among children below 15 years of age [4].

Although new HIV infections have been gradually declining globally, mother-to-child transmission (MTCT) of HIV remains a significant public health challenge. It contributes to early morbidity and mortality among children. Eliminating vertical HIV transmission is a global public health priority to improve maternal and neonatal health outcomes, extend life expectancy, and reduce the burden of chronic health conditions [5]. Treatment retention among PWHIV is also critical for preventing vertical HIV transmission [6].

WHO has defined criteria for validating the elimination of MTCT. These include achieving a population case rate of ≤ 50 new pediatric HIV infections per 100,000 live births and an MTCT rate of < 2% in non-breastfeeding populations or < 5% in breastfeeding populations. To meet these validation criteria, countries must sustain programmatic targets for at least two consecutive years. These targets include antenatal care (ANC) coverage (at least one visit) of ≥ 95%, HIV testing coverage among pregnant women of ≥ 95%, and ART coverage among PWHIV of ≥ 95% [6, 7]. In 2015, Cuba became the first country to achieve validation for eliminating vertical HIV transmission. Since then, several other countries, including Thailand, Belarus, Armenia, Anguilla, Montserrat, Cayman Islands, Bermuda, Antigua and Barbuda, St Kitts and Nevis, Malaysia, Maldives, Sri Lanka, Dominica, Oman, Belize, Jamica, Saint Vincent and Grenadines, had also achieved this milestone [8].

Nepal has a total population of 29,164,578 across its seven provinces. Of the 14,911,027 women in the country, 41% (6,145,039) are married women aged 15–49 years [9]. Around 94% of pregnant women had attended at least one ANC visits from skilled health personnel and 79% of the deliveries were conducted at health facilities [10]. There were 2,838 functional birthing centers across 77 districts [11].

Nepal is experiencing concentrated HIV epidemic among key populations with an estimated 30,300 PLHIV in 2023 and an adult HIV prevalence of 0.1% among those aged 15–49 years. There were approximately 1,100 children (aged 0–14 years) living with HIV, and fewer than 100 new infections were reported among children [12]. As of 2023, Nepal’s progress stood at 92% of PLHIV who knew their HIV status, 78% of PLHIV who received ART among those who knew their HIV status and 75% had suppressed viral load status of those who received the treatment against the global 95-95−95 targets [1].

Nepal launched its comprehensive Prevention of Mother-to-Child Transmission (PMTCT) program in February 2005, adopting a four-pronged strategy: preventing HIV infection among women of childbearing age, preventing unintended pregnancies among women living with HIV, preventing HIV transmission from mothers to their infants, and providing treatment, care, and support to PWHIV [13]. In 2013, Nepal expanded the PMTCT program with a community-based approach, expanding the services to all birthing centers and other community based maternal and neonatal health services with greater engagement of female community health volunteers, initially covering seven districts and subsequently scaling it up to all birthing centers, including private hospitals [14].

Nepal prioritizes the elimination of vertical HIV transmission through strategic actions, including universal HIV testing for pregnant women, access to ART for all PWHIV, and strengthened PMTCT program coordination with family health services [15]. This program is integrated with reproductive, maternal, and child health services. The Government of Nepal provides a wide range of free services to support effective MTCT prevention that includes HIV testing for pregnant women, ART for HIV-positive mothers, safe delivery services, early infant HIV testing, immediate treatment, postnatal counseling, follow-up, and community support, as detailed in the Nepal Safe Motherhood and Newborn Health Roadmap 2030 [16].

Despite two decades of implementing vertical HIV transmission elimination services in Nepal, no systematic assessment of the program’s implementation has been conducted. Such an evaluation is critical to optimizing the program and achieving the goal of eliminating vertical HIV transmission. This study aims to analyze the demographic and clinical characteristics, ART retention, and vertical transmission outcomes among women living with HIV who were pregnant between 2020 and 2023 in Nepal. The findings provide valuable insights into factors influencing HIV transmission risk and ART adherence.

## Methods

### Country Context

Nepal’s protocol advises at least four ANC visits during pregnancy for fetal assessments, birth counseling, iron/folic acid supplements, and free HIV screening [16]. The PMTCT services are integrated within the Maternal and Child Health (MCH) program [13]. Designated HIV testing and counseling centers employ a three-tier HIV testing algorithm, conforming to WHO standards and national guidelines, while primary health care centers and health posts use an initial determine test kit for screening [17]. We did not analyze data on the ANC coverage indicator because the most recent National Demographic and Health Survey (NDHS) has already been completed, providing the latest information on the country’s progress in ANC coverage [10]. Additionally, the routine health information system only reports aggregated ANC coverage data at the national level. Therefore, further analysis of this indicator was excluded.

Pregnant women identified as HIV-reactive through screening are referred to confirmatory HIV testing and linked to ART centers for care and treatment if diagnosed as HIV positive. ART is provided free of charge to pregnant women with HIV, along with antiretroviral (ARV) prophylaxis for exposed infants. Additionally, consented individuals receive follow-up reminders for ARV pill pick-up and early infant diagnosis (EID) testing via mobile health interventions [18].

### Study Design

This study employed a retrospective cohort design. The study was conducted using secondary data from national HIV care and ART tracking system, the national database of HIV program under Nepal government, that was used at the national level. Data were collected from 84 birthing centers which also provided ART services and supported EID for HIV-exposed children.

### Study Population

The study included all PWHIV who were registered in the national HIV care and ART tracking system.

### Study Period

The study covered pregnancies among women living with HIV occurring between 2020 and 2023, with follow-up continuing until July 2024.

### HIV Testing and Early Infant Diagnosis

HIV screening for pregnant women was conducted at ANC visits using rapid test kits, with reactive results confirmed via a three-tier algorithm. According to the 2020 guidelines, HIV-exposed infants underwent nucleic acid tests at birth, 4–6 weeks, and 9 months if prior tests were negative. Infants testing positive at any stage were started on ART immediately. A final serological test was done at 18 months or 3 months post-breastfeeding.

### Data Variables

The analysis also examined the influence of independent variables such as maternal age, geographic region, education, caste, employment, HIV risk group, place of delivery, WHO clinical stage, health facility type, ART status during pregnancy, ART regimen, baseline viral load, and time to ART initiation. Maternal age was categorized into < 25 years (young) and ≥ 25 years (older). Health facilities were grouped into municipality-based and metropolitan/sub-metropolitan-based categories. ART initiation was classified as “rapid” (within 7 days of HIV diagnosis) or “delayed” (> 7 days of HIV diagnosis). ART regimens were grouped by enzyme inhibition type: Non-nucleoside reverse-transcriptase inhibitors (NNRTI), Protease Inhibitor (PI), and Integrase strand transfer inhibitors (INSTI)-based regimens.

The primary outcome variables were:

1. **HIV Transmission Rate**: The proportion of HIV-exposed children diagnosed as HIV-positive, determined by dividing the number of positive NAT and serological results after 18 months or 3 months post-breastfeeding by the total number of HIV-exposed children.
2. **ART Coverage**: The proportion of HIV-positive exposed children who initiated ART.
3. **Retention in Treatment**: The proportion of PWHIV enrolled in PMTCT services who remained on treatment at 6, 12, and 24 months. Retention was defined as being “on treatment” at the end of the period. Women lost to follow-up (absence for > 28 days after a missed appointment), deceased, or who discontinued treatment were considered as not retained.

### Data Management and Analysis

Quantitative data from the HIV care and ART tracking system were exported to Excel. Co-authors working within the national HIV program extracted relevant electronic data from the national database according to standard guidelines for handling individual-level data, ensuring the exclusion of personal identifiers [18]. Data completeness and consistency were verified against hard copies from health facilities. Cleaned data were imported into SPSS version 22 for descriptive analysis, including frequency distributions, means, and medians. Further analysis, including chi-square tests and proportions with 95% confidence intervals, was conducted using STATA 18.

## Results

We analyzed data from 322 women living with HIV who became pregnant between 2020 and 2023 in Nepal (Table 1).

### Demographic Characteristics

The average age of the women was 26.9 years (Standard Deviation = 6.24), with the majority (56.2%) falling within the 25–39 age range. Educational attainment varied significantly: 39.4% of the women were illiterate, and only 1.6% had achieved a bachelor’s degree or higher. A large proportion (87.6%) were unemployed, and 42.5% had a monthly income at or below 97 USD. Caste distribution revealed that Janajati (30.4%) and Brahmin/Chhetri (23.0%) were the largest groups. The primary mode of HIV transmission was unsafe sexual behavior (66.8%) and 5.3% were the pregnant mother who had HIV through vertical transmission from their mothers. Geographically, the women were distributed across various provinces, with the highest representation from Madhesh (23.9%) and Bagmati (23.0%).

**Table 1:** Demographic characteristics of pregnant women living with HIV between 2020 and 2023 in Nepal (*n* = 322)

Demographic Characteristics	*N*	%
**Age (Mean**,** Median**,** SD)**	26.9, 24, 6.24	
**Age groups**		
15–19 years	26	8.1
20–24 years	104	32.3
25–39 years	181	56.2
40 + years	11	3.4
**Education**		
Illiterate	127	39.4
Primary	95	29.5
Secondary	71	22.0
Higher secondary	24	7.5
Bachelor and higher	5	1.6
**Employment**		
Unemployed	282	87.6
Employed	40	12.4
**Average Monthly Income**		
≤ 97 USD (NPR 13,000)	17	42.5
> 97 USD (NPR 13,000)	17	42.5
did not want to share	6	15.0
**Caste**		
Janajati	98	30.4
Brahmin/Chhetri	74	23.0
Madhesi	69	21.4
Dalit	61	18.9
Others	20	6.3
**Possible transmission mode**		
Unsafe sexual behavior	215	66.8
Others (does not want to disclose)	87	27.0
Vertical Transmission	17	5.3
Drug injection	3	0.9
**Province**		
Koshi	53	16.5
Madhesh	77	23.9
Bagmati	74	23.0
Gandaki	19	5.9
Lumbini	59	18.3
Karnali	10	3.1
Sudurpaschim	30	9.3

### Clinical Characteristics

Table 2 outlines the clinical characteristics of the participants. A substantial proportion (44.1%) were diagnosed with HIV before 2020, 10.6% of PWHIV delivered babies at home and 58.1% received their diagnosis prior to pregnancy. At the time of diagnosis, most women were classified as WHO clinical Stage 1 (75.5%). Regarding ART initiation, 34.8% started ART on the same day as their diagnosis, while 40.1% initiated therapy within one week. During pregnancy, 56.8% were already on ART. Most women (91.6%) were on an INSTI-based regimen at the time of reporting, and 86.3% had a viral load below 1000 copies/mL.

In terms of ARV prophylaxis, 61.5% of the babies received Nevirapine (NVP) alone, while 3.7% did not receive any prophylaxis.

**Table 2:** Clinical characteristics of pregnant women living with HIV between 2020 and 2023 in Nepal (*n* = 322)

Clinical Characteristics	*N*	%
**Year of HIV diagnosis**		
< 2020	142	44.1
2020	22	6.8
2021	50	15.5
2022	73	22.7
2023	35	10.9
**Place of Delivery**		
Home	34	10.6
Health facility	288	89.4
**HIV diagnosis status**		
HIV diagnosed at the time of pregnancy	135	41.9
HIV diagnosed before pregnancy	187	58.1
**WHO clinical stages at diagnosis**		
Stage 1	243	75.5
Stage 2	59	18.3
Stage 3 and Stage 4	20	6.2
**Time to initiate ART**		
Same day initiation	112	34.8
Rapid initiation (within 1–7 days of HIV diagnosis)	129	40.1
Late initiation (> 7 days of HIV diagnosis)	81	25.1
**ART status in pregnancy**		
Already on ART	183	56.8
During pregnancy	139	43.2
**Category of ARV regimen at initiation**		
INSTI-based regimen with NRTIs	180	55.9
NNRTI-based regimen	141	43.8
PI based regimen	1	0.3
**Current category of ARV regimen**		
INSTI-based regimen with NRTIs	295	91.6
NNRTI-based regimen	26	8.1
PI based regimen	1	0.3
**Viral load at diagnosis**		
< 1000 copies/mL	278	86.3
1000 or more copies/mL	15	4.7
Had not tested for VL	29	9.0
**ARV prophylaxis for baby**		
Nevirapine	198	61.5
Zidovudine + Nevirapine	112	34.8
Not received	12	3.7

Note: Same day initiation: ART initiation at the same day of HIV diagnosis with prescribed ART; Rapid ART initiation: ART initiated within 1–7 days of HIV diagnosis; INSTI: Integrase Strand Transfer Inhibitor; NRTI: Nucleoside Reverse Transcriptase Inhibitors; NNRTI: Non-nucleoside Reverse Transcriptase Inhibitors; PI: Protease inhibitor

### Retention in Care

Retention rates were notably high across all time points: 96.9% at 6 months, 94.8% at 12 months, and 96.0% at 24 months (Table 3).

Retention was consistent across age groups, with women aged 25 and above achieving rates of 96.4% at 6 months, 94.1 at 12 months and 95.5% at 24 months. Minimal variation was observed by education and employment status, with illiterate women showing a retention rate of 96.1% at 6 months and unemployed women at 96.8%. Retention rates across caste groups were comparable, although Dalit women demonstrated lower retention at 6 months (93.4%), at 12 months (89.8%) and at 24 months (90.5%) in comparison to women belongs to other caste groups. When stratified by transmission mode, retention was high among those with HIV acquired through unsafe sexual behavior (97.2% at 6 months), while all cases of vertical transmission had 100% retention rate at all points of time. Retention rates were uniformly high across health facility types and ARV regimens. Women on INSTI-based + NRTI regimens maintained retention rates of 96.6% at 6 months and 95.5% at 24 months.

**Table 3:** Retention in treatment of pregnant women living with HIV between 2020 and 2023 in Nepal

Subgroups	Still on ART at 6 months		Still on ART at 12 months		Still on ART at 24 months	
	*N*	% (95% CI)	*N*	% (95% CI)	*N*	% (95% CI)
**Overall**	312	96.9 [94.3–98.4]	294	94.8 [91.7–96.8]	241	96.0 [92.7–97.8]
**Age groups**						
15–24 years	127	97.7 [93.1–99.3]	119	96.0 [90.6–98.3]	92	96.8 [90.6–99.0]
25 + years	185	96.4 [92.5–98.3]	175	94.1 [89.6–96.7]	149	95.5 [90.8–97.9]
**Education**						
Illiterate	122	96.1 [90.9–98.4]	117	92.9 [86.8–96.3]	108	95.6 [89.8–98.2]
Literate	190	97.4 [94.0–98.9]	177	96.2 [92.2–98.2]	133	96.4 [91.5–98.5]
**Employment**						
Unemployed	273	96.8 [94.0–98.3]	258	94.9 [91.5–96.9]	215	96.0 [92.4–97.9]
Employed	39	97.5 [84.1–99.7]	36	94.7 [81.1–98.7]	26	96.3 [77.7–99.5]
**Caste**						
Janajati	95	96.9 [90.9–99.0]	89	94.7 [87.8–97.8]	75	98.7 [91.1–99.8]
Brahmin/Chhetri	72	97.3 [89.8–99.3]	68	97.1 [89.2–99.3]	55	94.8 [85.1–98.3]
Madhesi	68	98.6 [90.3–99.8]	65	97 [88.8–99.3]	58	98.3 [88.8–99.8]
Dalit	57	93.4 [83.7–97.5]	53	89.8 [79.1–95.4]	38	90.5 [77.1–96.4]
Others	20	100	19	95 [71.6–99.3]	15	93.8 [66.1–99.1]
**Possible transmission mode**						
Unsafe sexual/injecting behaviour	212	97.2 [94.0–98.8]	198	95.2 [91.3–97.4]	162	95.3 [90.8–97.6]
Others (does not want to disclose)	16	95.4 [88.3–98.3]	79	92.9 [85.1–96.8]	62	96.9 [88.2–99.2]
Vertical Transmission	83	100	16	100	16	100
**Health facility based at**						
Municipality	110	99.1 [93.8–99.9]	101	96.2 [90.2–98.6]	80	93.0 [85.3–96.8]
Metropolitan and sub metropolitan	202	95.7 [92.0–97.8]	193	94.1 [89.9–96.7]	161	97.6 [93.7–99.1]
**Time of HIV diagnosis**						
At the time of pregnancy	127	94.1 [88.6–97.0]	112	91.1 [84.5–95.0]	63	94.0 [85.1–97.8]
Before pregnancy	185	98.9 [95.8–99.7]	182	97.4 [93.7–98.9]	178	96.7 [92.9–98.5]
**Time to initiate ART**						
Within 7 days of HIV diagnosis	232	95.3 [90.0–97.9]	219	95.3 [89.2–98.1]	170	97.3 [89.6–99.3]
> 7 days of HIV diagnosis	80	98.8 [91.7–99.8]	75	97.4 [90.1–99.4]	71	97.3 [89.6–99.3]
**ART status in pregnancy**						
Already on ART	131	94.2 [88.9–97.1]	114	89.8 [83.1–94.0]	64	92.8 [83.7–97.0]
During pregnancy	181	98.9 [95.7–99.7]	180	98.4 [95.0–99.5]	177	97.3 [93.5–98.9]
**WHO clinical stages at Dx**						
Stage 1	233	95.9 [92.5–97.8]	216	93.5 [89.5–96.1]	170	95.5 [91.2–97.7]
Stage 2	59	100	58	98.3 [88.8–99.8]	52	98.1 [87.6–99.7]
Stage 3 and Stage 4	20	100	20	100	19	95.0 [71.5–99.3]
**Current ARV regimen**						
INSTI-based + NRTIs	285	96.6 [93.8–98.2]	267	94.3 [90.9–96.5]	214	95.5 [91.9–97.6]
NNRTI and PI based regimen	27	100	27	100	27	100
Viral load at diagnosis*						
< 1000 copies/mL	273	98.2 [95.7–99.3]	258	96.3 [93.2–98.0]	214	96.4 [92.9–98.2]
1000 or more copies/mL	15	100	15	100	12	92.3 [60.6–98.9]

Note: *Excluded missing value of those who did not have VL test

### Vertical Transmission Rates

**Fig. 1:**
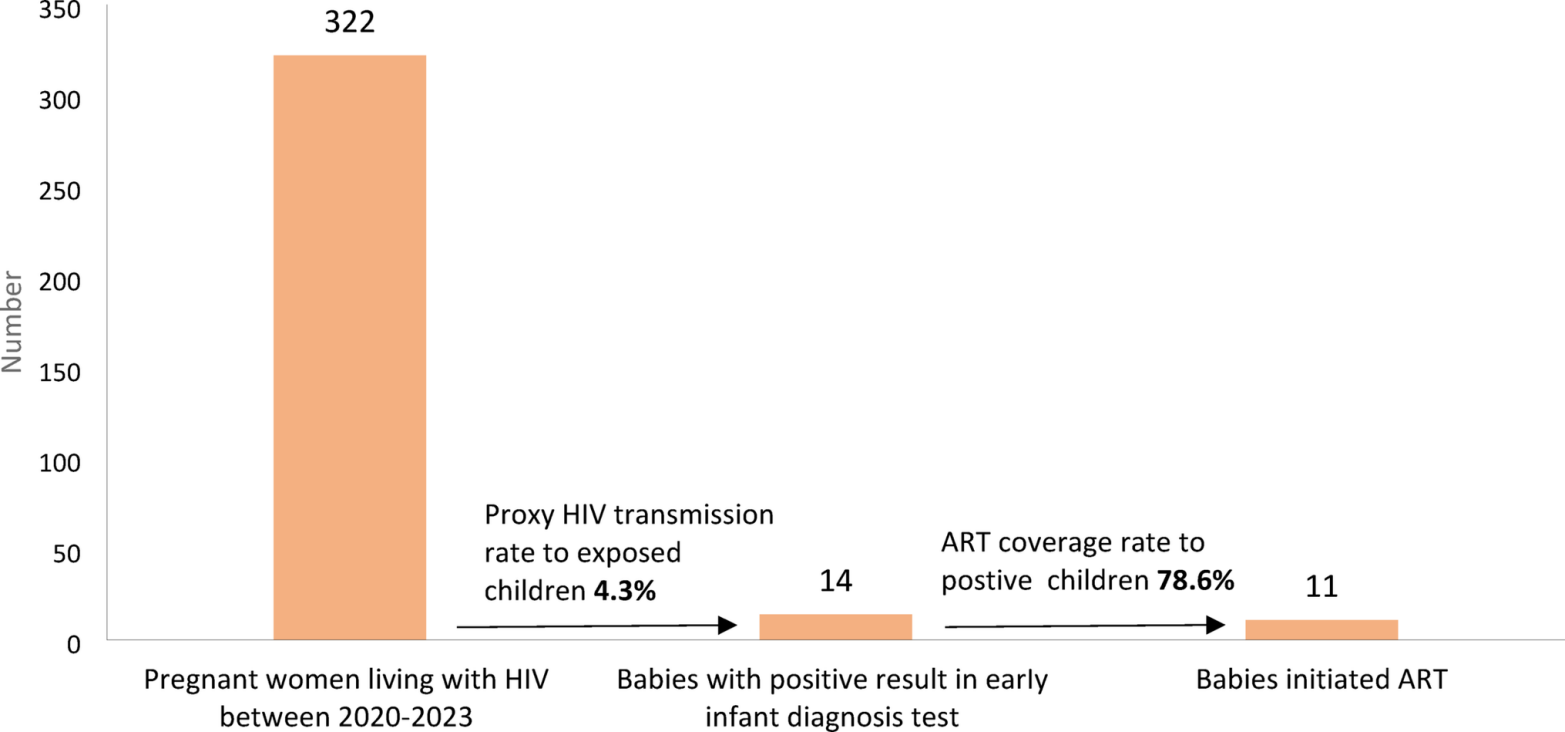
Pregnant women living with HIV and outcomes of their delivered child between 2020 to 2023

As illustrated in Fig. 1; Table 4, the overall vertical transmission rate was 14 out of 322 pregnancies, corresponding to 4.3% (95% CI 2.4 to 7.2), with ART coverage among infected infants at 78.6% (11 out of 14 infants born with HIV).

Transmission risk varied by maternal age, with mothers aged 25 years or older exhibiting a lower transmission rate (2.6%) compared to younger mothers (6.9%). Variations were also observed by education and employment status, with illiterate mothers showing a transmission rate of 5.5% and unemployed mothers 5.0%. Among caste groups, Dalit mothers experienced the highest transmission rate (11.5%). The highest transmission rate, 17.6%, was observed in pregnancies where delivery occurred at home.

Higher transmission rates were associated with certain clinical factors. Infants born to women diagnosed at WHO clinical Stage 3 or 4 had a transmission rate of 11.1%. Similarly, women diagnosed with HIV during pregnancy (8.9%), those who started ART during pregnancy (8.6%), and those with a viral load of 1000 copies/mL or higher at the time of diagnosis (13.3%) showed elevated risks of vertical transmission.

**Table 4:** Vertical transmission risk and ART coverage among children born between 2020 and 2023 in Nepal (*N* = 14)

Mothers’ subgroups	Transmission risk of HIV to their child		ART coverage of infected child	
	*N*	%	*N*	%
**Overall**	14	4.3	11	78.6
**Age groups**				
15–24 years	9	6.9	7	77.8
25 + years	5	2.6	4	80.0
**Education**				
Illiterate	7	5.5	5	71.4
Literate	7	3.6	6	85.7
**Employment**				
Unemployed	14	5.0	11	78.6
Employed	0			
**Caste**				
Janajati	3	3.1	3	100
Brahmin/Chhetri	3	4.1	2	66.7
Madhesi	1	1.4	1	100
Dalit	7	11.5	5	71.4
**Possible transmission mode**				
Unsafe sexual/injecting behaviour	11	5.1	8	72.7
Others (does not want to disclose)	2	2.3	2	100
Vertical Transmission	1	5.9	1	100
**Health facility based at**				
Municipality	9	7.3	7	77.8
Metropolitan and sub metropolitan	5	2.5	4	80
**WHO clinical stages at diagnosis**				
Stage 1	10	4.1	8	80
Stage 2	2	3.4	2	100
Stage 3 and Stage 4	2	11.1	1	50
**Time of HIV diagnosis**				
At the time of pregnancy	12	8.9	10	83.3
Before pregnancy	2	1.1	1	50
**ART status in pregnancy**				
Already on ART	12	1.1	10	83.3
During pregnancy	2	8.6	1	50
**Place of Delivery**				
Home	6	17.6	6	100
Health facility	8	2.8	5	62.5
**Time to initiate ART**				
Within 7 days of HIV diagnosis	2	5	2	100
> 7 days of HIV diagnosis	12	2.5	9	75
**Current of ARV regimen**				
INSTI-based + NRTIs	14	4.7	11	78.6
NNRTI and PI based regimen	0			
Viral load at diagnosis*				
< 1000 copies/mL	9	3.2	7	77.8
1000 or more copies/mL	2	13.3	1	50
**Status of ARV prophylaxis**				
Completed	4	2.1	3	75
Not completed	10	7.5	8	80

Note: *Excluded missing value of those who did not have VL test

## Discussion

Our study highlights key findings related to the prevention of vertical HIV transmission in Nepal. Six out of ten women living with HIV were diagnosed before pregnancy, with nearly half diagnosed before 2020. At diagnosis, the majority were in WHO clinical Stage 1, and more than half were already on ART during pregnancy. ART initiation timing varied, with a substantial proportion starting treatment either on the same day or within one week of diagnosis. Most women were prescribed INSTI-based regimens, and the majority had viral loads below 1000 copies/ml. Retention on ART was high across all time points (6, 12, and 24 months) and remained consistent across demographic groups, with only slight variations. The overall vertical transmission rate was low (4.3%), and ART coverage among HIV-infected infants was 78.6%. However, higher transmission risks were observed among younger mothers, Dalit women, those diagnosed with advanced-stage HIV, those diagnosed or initiating ART during pregnancy, and those with higher viral loads at diagnosis. The highest transmission rate, 17.6%, was observed in pregnancies where delivery occurred at home.

This study provides a comprehensive programmatic analysis of efforts to eliminate vertical HIV transmission in Nepal. The ANC coverage from skilled providers increased from 86% in 2016 to 94% in 2022  [10], it remains slightly below the WHO benchmark. Similarly, HIV testing coverage among pregnant women has shown improvement, reaching 96% in 2022/2023 [12, 14]. However, aggregated data reporting from ANC services lacks a robust monitoring mechanism to prevent duplication, raising concerns about data quality. If Nepal sustains its current HIV testing coverage, it is likely to meet the testing criterion for elimination of mother-to-child transmission of HIV.

The coverage of pregnant women receiving ARVs for PMTCT could not be determined directly from the HIV Care and ART Tracking System, as only those who initiated ART were included in the database and it does not cover those PWHIV who never initiated treatment. Global AIDS Monitoring reports indicated stable coverage, at 83% in 2022 and 80% in 2023 [19]. For the establishment of the robust monitoring mechanism there is a need to scaling up the existing database system to record PWHIV diagnosed at all ANC service sites and tracking those who could not initiate ART for developing the tailored intervention to address the gap. Approximately 60% of PWHIV in our study became pregnant after their HIV diagnosis, reflecting higher desire to get pregnant and fertility trends similar to those reported in Thailand, Rwanda and Ethiopia [20–23]. Therefore, the family planning services should be integrated with the ART services to deliver the counseling sessions to the women living with HIV along with their spouse for different types of contraceptives methods to use and prevent the unwanted pregnancies and also support to those who wants conception to bear a child.

Retention rates among PWHIV in our study were consistently high (≥ 95%) at 6, 12, and 24 months, exceeding the national retention rates and those reported in a study from south India, Indonesia and several African countries [12, 24–26]. The overall treatment retention rate at 6 months was similar to the developed nation Switzerland however it is observed lower among the Dalit women. The proxy HIV transmission rate to exposed children was 4.3% in this study, which is below the WHO target of < 5% for elimination of vertical transmission [6] and lower than rates reported in India (8.76%) [27]. The transmission rate was varying with demographic variables. However, variations in transmission rates were observed across demographic groups. Younger mothers (< 25 years) experienced higher transmission rates compared to older mothers (≥ 25 years), aligning with findings from Uganda [28]. Similarly, illiterate mothers had a higher transmission rate, consistent with studies conducted in Karnataka, India [29].

Unemployed and Dalit women also exhibited elevated transmission rates due to inequalities in accessing and utilization of the health services which was also observed in some other health service utilization studies conducted in Nepal [30, 31]. Dalits, historically marginalized and labeled “untouchables” in the Hindu caste system by so called higher castes, such as Brahmins or Chhetris, faced severe discrimination. Despite recent legal changes, they still experience reduced opportunities in healthcare and education due to decades of oppression and discrimination [32–34]. This systemic marginalization has perpetuated a vicious cycle of poverty across multiple generations. The high transmission rate of HIV among children born to Dalit women living with HIV can be attributed to the structural violence they have experienced for generations, rooted in both societal and institutional discrimination in Nepal. Late HIV diagnosis during pregnancy and delayed ART initiation were significant risk factors for vertical transmission, as corroborated by findings from Kenya [35].

Transmission was more likely among children who did not complete ARV prophylaxis, consistent with studies in Ethiopia [36]. Gaps in ARV treatment enrollment were noted, particularly among children born to illiterate, unemployed, underprivileged, and marginalized caste groups, as well as those residing in remote areas.

The strengthening of the tracking and surveillance system was one of the major attributes in achieving eMTCT in Thailand and Sri Lanka [37]. To enhance programmatic decision-making, Nepal must prioritize strengthening its data systems to ensure the availability of quality evidence. Improved mechanisms for identifying gaps at the provincial, district, and facility levels are essential.

Nepal has demonstrated progress in reducing vertical transmission of HIV and increasing HIV testing among pregnant women. However, targeted efforts are needed to address disparities among specific sub-groups, including young, illiterate, unemployed, and marginalized women, particularly those residing in remote areas such as Karnali and Sudurpaschim provinces. To achieve eMTCT, Nepal must sustain its current achievements while focusing on improving ANC coverage, ART coverage among pregnant women, and infant ARV prophylaxis coverage.

Unlike to those countries who had successfully validated for eMTCT, strong political leadership and commitment for eMTCT is required for continuing advocacy at the national, provincial and district level in Nepal [37–39]. Nepal should further enhance strengthening the integration of PMTCT services within maternal, newborn, child and adolescent health programs beyond the facility-based services. Nepal should learn from the experience of Thailand about the health insurance scheme to improve integration of PMTCT services with maternal and child health services [37]. There is a universal HIV testing policy among pregnant women, however attention should also be given to promote and ensure institutional deliveries of all PWHIV with massive engagement of the community health workers in collaboration of non-government system. Robust community-based monitoring and tracking mechanisms for PWHIV and their children should be established mobilizing the community health workers of government in collaboration with non-government systems to bridge treatment gaps. Maldives has an excellent example of coordination and collaboration of public and private health facilities for supporting eMTCT [40]. Similarly, Nepal should also build a strong mechanism to mainstream the private health facilities to the national health system with expanding private-sector engagement in reporting systems. Furthermore, the prevention service improvements should include scaling up pre-exposure prophylaxis for discordant couples, radio jingles and other mass media awareness on PMTCT, and fostering coordination with community stakeholders, including female community health volunteers. Efforts should also focus on addressing gaps in the ART initiation process, particularly among pregnant women, and enhancing laboratory networks to support early infant diagnosis. The current reliance on centralized testing facilities, such as the National Public Health Laboratory, necessitates scaling up provincial laboratories and optimizing sample transport systems for dried blood spot testing.

To our knowledge, this is the first national analysis of Nepal’s PMTCT program aimed at guiding elimination of mother-to-child transmission of HIV strategies. However, the study had limitations. First, the analysis was constrained by the absence of certain key variables, such as gestational age, gravidity, breastfeeding practices, and infant’s sex. Second, the unavailability of data on those pregnant women who were diagnosed and never enrolled on ART in the existing HIV care ad ART tracking system hindered a comprehensive assessment of vertical transmission rates for all children born to the PWHIV.

In conclusion, while Nepal is on the path toward eliminating vertical transmission of HIV, sustained investments in health systems, equitable access to services, and quality data will be essential to meet this critical public health milestone.

## Data Availability

Data will be made available upon request made to the corresponding author.
